# An Adaptive Force Matching Potential for Alanine Developed with Møller–Plesset Perturbation Theory and Smooth Fourier Transform Correction Map

**DOI:** 10.1021/acs.jpcb.5c04529

**Published:** 2025-08-26

**Authors:** Ying Yuan, Feng Wang

**Affiliations:** Department of Chemistry and Biochemistry, 3341 University of Arkansas, Fayetteville, Arkansas 72701, United States

## Abstract

Developing accurate force fields for biomolecules remains a significant challenge due to the subtle energetic differences between various conformational states. We present a novel force field model for polyalanine, ALAMP2_25, developed using adaptive force matching (AFM) with Møller–Plesset perturbation theory at the second order (MP2) as the reference method. By fitting smaller model compounds and transferring parameters to larger peptides, we overcome the limitations of traditional AFM approaches and enable the use of more accurate electronic structure methods. The ALAMP2_25 model incorporates a new correction scheme, Smooth Fourier Transform-based φ, ψ correction map (SFT-CMAP), which efficiently describes φ, ψ coupling with reduced overfitting. Our model demonstrates good agreement with experimental *J*-coupling data for hydrated polyalanine and shows improved transferability to *N*-methylated cyclic alanine when compared to previously reported DFT based models. The developed framework provides a pathway for creating accurate force fields for a broader range of amino acids and biomolecules, enabling first-principles-based simulations of complex biological systems with applications in protein folding, ligand binding, and drug design.

## Graphical abstract

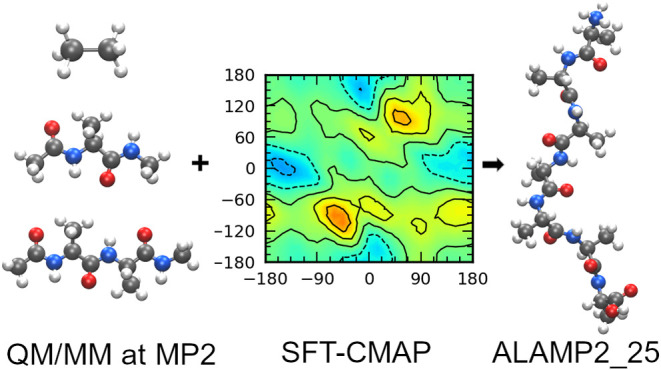

## 1 Introduction

Developing accurate force fields for peptides and proteins remains a significant challenge due to the subtle energetic differences between various conformational states. Many existing force fields are empirically adjusted to stabilize prevalent structures found in the Protein Data Bank (PDB). While these models have enabled high-quality simulations and advanced the field of computational biology,^1–10^ they exhibit known deficiencies. For instance, traditional empirical protein force fields often struggle to accurately describe less common protein folds or intrinsically disordered peptides in hydrated environments.^11–17^

Recently, attempts have been made to fit force fields only to electronic structure calculations without allowing for empirical adjustments.^18–22^ For examples, models developed using adaptive force matching (AFM),^23–25^ have demonstrated excellent agreement with experiments for a few peptides.^24,25^ However, the AFM method typically requires fitting to relatively long peptide segments, limiting its scalability to computationally affordable methods like density functional theory (DFT).

Despite the success of DFT, it is well-known that most popular functionals suffer from charge-delocalization errors,^26–30^ leading to an overestimation of polarization stabilization. Recent studies have demonstrated that polarization stabilization plays a crucial role in determining the conformational stabilities of poly peptides.^31^ Such a strong role of polarization is a result of different secondary structures exhibiting distinct dipole alignments. For example, α-helices are stabilized by favorable dipole alignment, whereas β-strands experience polarization frustration due to the antiparallel alignment of amide dipoles, which makes it challenging for water molecules to stabilize adjacent local dipoles of opposite orientations.^31^

In this study, we aim to develop a more accurate force field model for alanine using the Møller–Plesset perturbation theory at the second order (MP2).^32^ MP2 is anticipated to provide a more accurate description of electron correlation in molecules with large band gaps, such as proteins, relative to commonly employed density functionals. A key advantage of MP2 is that it is less likely to overestimate the importance of polarization effects.^28^ While many DFT functionals significantly overestimate the strength of hydrogen bonds, leading to a freezing temperature of liquid water that is too high,^33^ it has been shown that MP2 provides a high-quality description of the potential energy surface of water.^34–36^ When fitted through AFM, MP2 has been found to give excellent hydration free energies of small organic molecules.^37,38^

To enable the use of MP2, we depart from the traditional AFM approach by fitting smaller model compounds and transferring the parameters to larger peptides. This new approach makes it possible to develop force fields for a broader range of amino acids at a lower computational cost. Additionally, we introduce a Fourier transform-based φ, ψ correction map (CMAP) that uses fewer parameters than traditional grid-based CMAPs,^6,8^ reducing the risk of overfitting.

Our MP2-based alanine model demonstrates good agreement with experimental *J*-coupling data^39^ for hydrated polyalanine and provides reasonable performance when applied to *N*-methylated cyclic polyalanine^40^ without refitting any parameters, outperforming a previously developed DFT-based model. This study shows that parameters fitted with AFM using small model compounds can be transferred to much larger molecules with similar bond connectivity. The success paves the way for the development of more accurate force fields for a wider range of amino acids and other macromolecules.

The paper is organized into four sections. Following the introduction, we outline the model compound-based fitting procedure and the Fourier Transform-based CMAP methodology in the Methods section. The Results and Discussion section reports the performance of our model in simulating hydrated cationic peptides and its transferability to *N*-methylated cyclic-peptides. The final section summarizes the key findings and conclusions.

## 2 Methods

### 2.1 Model Compound-based Fitting of AFM Models

The Adaptive Force Matching (AFM) method is an iterative force field development approach consisting of three steps.^23,24,37,41–44^ The first step involves sampling, where the current force field is used to generate a training set relevant to the system and thermodynamic conditions of interest. This is followed by quantum mechanics/molecular mechanics (QM/MM) calculations to obtain reference forces, which are then used in the force matching (FM)^45,46^ step to refine the force field. The refined force field will then be used to initiate a new iteration of the three steps. The iterative process ensures high-quality sampling and an accurate MM model for the QM/MM calculations.

The details of our model-compound-based fitting of the alanine model using MP2 reference are provided in the Supporting Information. This section highlights key decisions made during the fitting of the model.

To reliably capture solute-water interactions at the QM level, AFM employs a large QM region that encompasses many hydration waters in the QM/MM step. Furthermore, current AFM models do not prioritize transferability and generally assign unique atom types to all atoms that are not equivalent by symmetry. Consequently, fitting a poly peptide model would require a very large QM region to ensure coverage of all unique atom types, which limits the QM methods that can be used to provide reference forces.

For example, our previous DFT-based alanine model^24^ fitted (Ala)_7_ with up to 106 hydration waters in the QM region. Such a large QM region makes it impractical to use MP2 as a reference method, as MP2 gradient calculations require solving the coupled-perturbed Hartree–Fock equations. It scales formally as *N* ^5^,^47,48^ although algorithms exist to mitigate this scaling.^49,50^ In this study, to enable the use of the computationally expensive MP2 method, we fit only alanine dipeptides to determine all peptide-water parameters and use alanine tripeptides to determine intrapeptide parameters.

We believe that modeling each symmetry-unique atom as a distinct atom type is not strictly necessary in AFM, and that the same pair-specific parameters can be used for sufficiently similar atom pairs.^51^ This is a reasonable approximation as long as the force field accurately captures the underlying physics, rather than relying on extensive error cancellations. This approach reduces numerical challenges in the fitting process and allows more accurate electronic structure methods to be used as reference.

#### 2.1.1 Dipeptide-Based Fit of Intermolecular Parameters

To determine the peptide-water parameters, 320 conformations of the blocked alanine dipeptide are sampled from short molecular dynamics (MD) simulations in each generation of AFM. The QM region comprises a blocked alanine dipeptide (Figure 1a) and its hydration water. In addition, the QM region consists of a fitting zone and a buffer zone. The buffer zone is designed to eliminate close contacts between MM and fitting zone atoms. Only forces on atoms within the fitting zone are used to train the force field. In this step, the peptide and at least five hydration shell water molecules are selected for inclusion in the fitting zone. The remaining first hydration shell water and additional water molecules within 2.6 Å of the fitting zone water molecules are included in the buffer zone. The def2-TZVP basis set^52^ is used for atoms in the fitting zone, while the def2-SVP basis set^52^ is used for atoms in the buffer zone.

**Figure 1:**
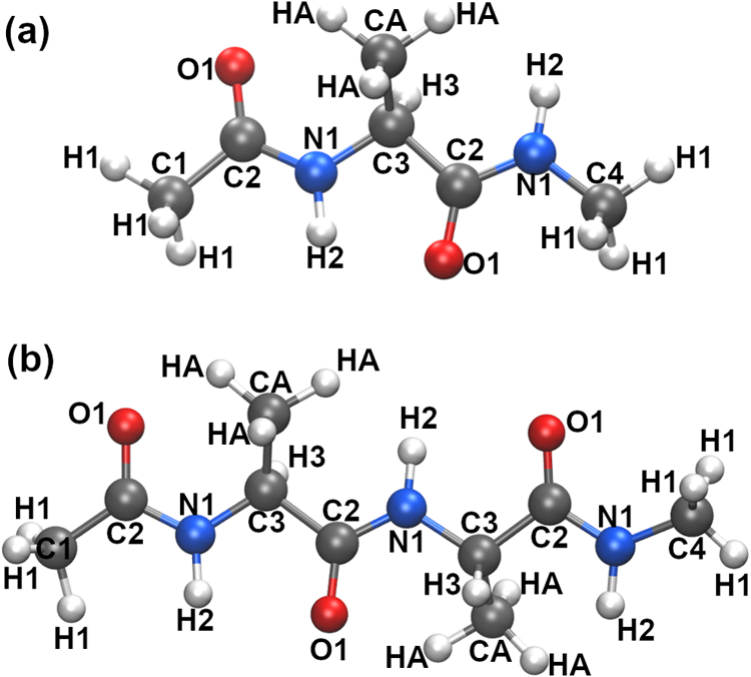
Dipeptide and tripeptide structures used for fitting the peptide-water intermolecular and peptide-intramolecular parameters. Atom types are labeled.

As reported recently by our group, modeling nonpolar molecules without partial charges leads to better parameter reliability without compromising the model’s accuracy in reproducing thermodynamic properties.^53^ Therefore, we do not assign partial charges to nonpolar groups. Only the four atoms of the amide group carry partial charges and are constrained to be neutral. We note that the partial charges in AFM were fitted indirectly using charge products, as the latter are linear parameters of the forces.^23,44^ Fitting charge products therefore provide improved numerical stability.

#### 2.1.2 Tripeptide-Based Fit of Intramolecular Terms

Following the fitting of peptide-water interactions, we perform QM/MM calculations using blocked tripeptides (Figure 1b) to determine intramolecular terms, including harmonic bond, harmonic angle, cosine torsional, and short-range nonbonded interactions. Due to the short length of the tripeptide, side chain-side chain steric repulsion is not adequately sampled during the MD step. Therefore, the methyl–methyl side chain nonbonded interaction is taken from an MP2-based ethane model developed previously with AFM.^53^

In each generation of AFM, 500 tripeptide conformations were sampled. Since only intramolecular terms are being fit, only the peptide is included in the fitting zone of the QM region. All water molecules within the first hydration shell of the peptide are modeled as QM particles in the buffer zone. The peptide is described using the def2-TZVP basis set, while all buffer zone molecules are described using the def2-SVP basis set.

A total of seven generations of AFM were performed, and the last four generations were used as the global set to fit the final force field. The dispersion was fitted prior to the AFM iterations using small molecular fragments with symmetry-adapted perturbation theory (SAPT).^38,54^ The correction map (CMAP) is fitted only once using this global set after the model without CMAP was iteratively developed.

### 2.2 Smooth Fourier Transform (SFT)-CMAP

While many protein force fields employ a grid-based CMAP to model φ, ψ coupling,^6,8^ the AFM-based ALA2022 model achieved good agreement with experimental *J*-coupling constants without explicit consideration of such φ, ψ coupling.^31^ Figure 2 compares the potential energy surface (PES) of the peptide as a function of φ, ψ computed with the AFM model without CMAP correction and the MP2 peptide-only energy (*E*_pep_) surface computed with the conductor-like polarizable continuum model (CPCM).^55–59^

**Figure 2:**
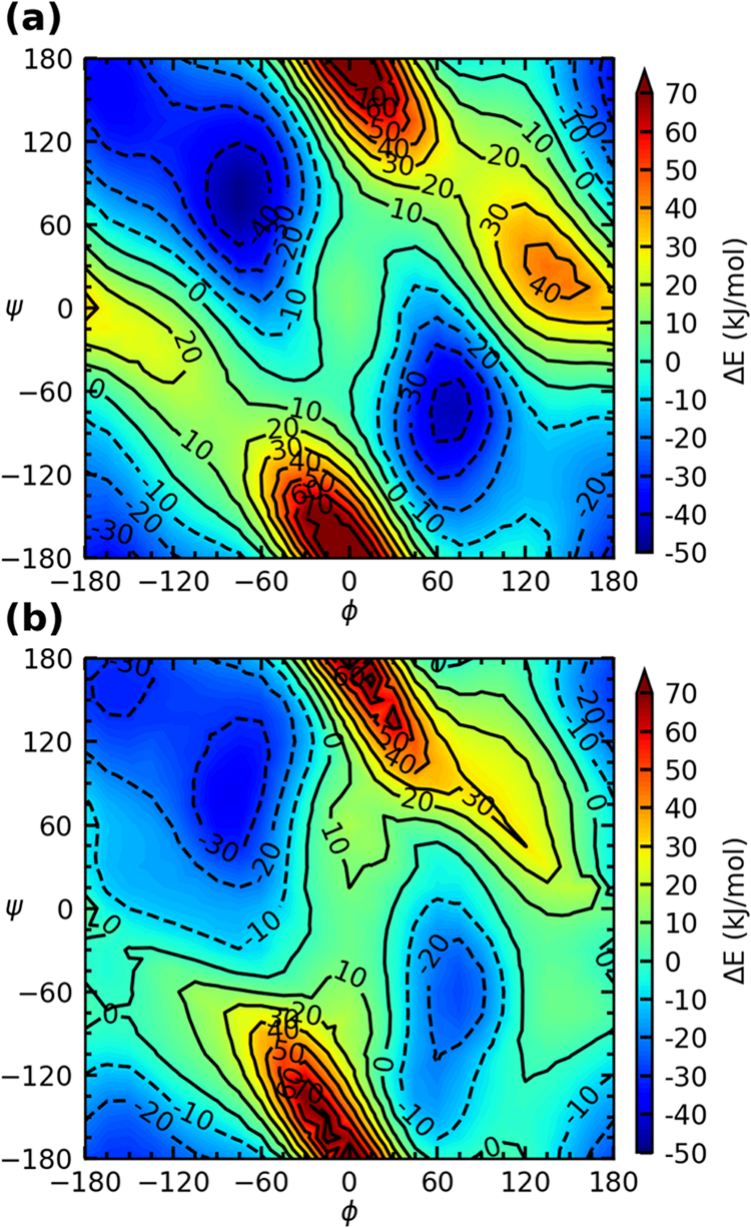
PES of the blocked dipeptide computed with (a) the ALAMP2_25 model without SFT-CMAP and (b) the *E*_pep_ surface from MP2 CPCM calculations.

It is important to note that the CPCM energies calculated with MP2 are not directly comparable to force field energies, as the CPCM method includes the contribution from solvent–water interactions. To achieve a more appropriate comparison, the solvent–water interaction energy, *E*_diel_, is subtracted from the CPCM energy to obtain the CPCM peptide-only energy, as shown in the following equation

1 $$E_{pep}=E_{MP2\_\mathrm{CPCM}}-E_{diel}$$

where *E*_MP2_CPCM_ is the CPCM energy in an implicit solvent of water and *E*_diel_ is the CPCM dielectric energy. The resulting *E*_pep_ represents the best approximation of the peptide-only conformational energy inside an implicit solvent of water.

Comparing the two PESs, the regions with the largest difference are (165, 15) and (0, −180). The former corresponds to the conformation where the amino hydrogens of the two amides approaches each other, whereas the latter corresponds to the conformation where the two carbonyl oxygens face each other (Figure S1). It is worth noting that the partial charges of the MP2 were derived solely from fitting peptide-water interactions. Thus, it is expected that such interactions are not sufficiently accurate to describe peptide–peptide Coulombic interactions. In cases where two adjacent dipoles are opposing each other, induction effects are expected to reduce the dipole moments so as to reduce dipole–dipole repulsion. Because only point-charge models are being fitted in this study, there is no mechanism to reduce this repulsion. Consequently, this effect is best captured with CMAP.

We note that typical AFM force fields ignore nonbonded interactions for 1–2 and 1–3 atom pairs but fully consider them for 1–4 atom pairs, which are separated by three covalent bonds. However, the dipole–dipole interaction between the highlighted atoms in Figure 3a involves 1–3, 1–4, and 1–5 Coulombic interactions. Neglecting 1–3 interactions would miss a significant component of this dipole–dipole interaction. Although empirical scaling of 1–4 interactions might alleviate this problem, it would effectively circumvent the issue through error cancellations. Furthermore, Figure 3b highlights dipole–dipole interactions involving a combination of 1–4, 1–5, and 1–6 interactions, and scaling 1–4 interactions would introduce inaccuracies in modeling these interactions. One way to address the problem associated with appropriate modeling of such adjacent dipole moments on the peptide is CMAP.

**Figure 3:**
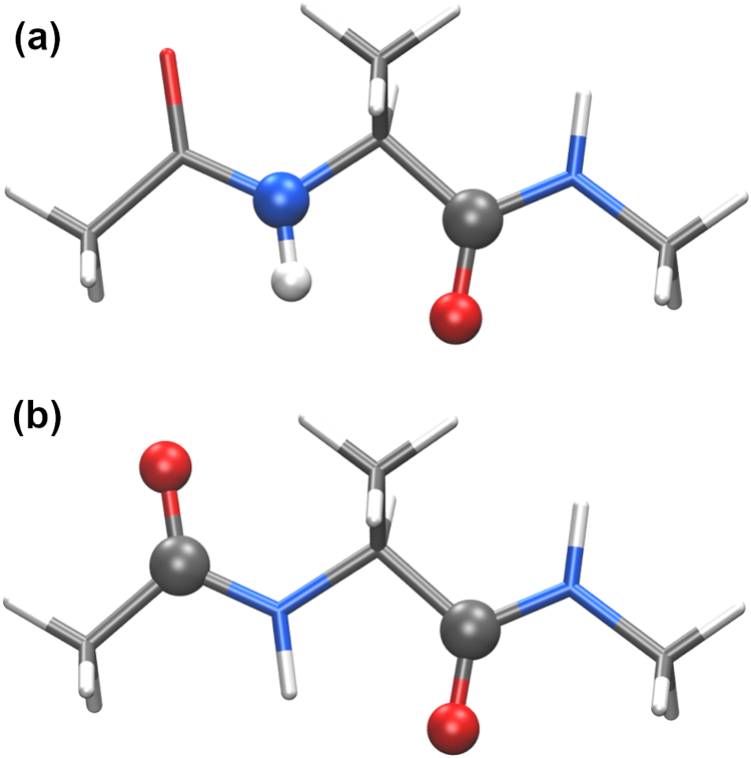
Schematic illustration of dipole–dipole interactions between adjacent polar groups, involving (a) 1–3, 1–4, and 1–5 Coulombic interactions, and (b) 1–4, 1–5, and 1–6 Coulombic interactions.

Specifically, we utilize a new variant of CMAP, termed Smooth-Fourier-Transform (SFT)-CMAP. SFT-CMAP represents the energy correction as a function of φ and ψ angles using a truncated 2D Fourier Transform. The energy at each grid point, *E_S_* (ϕ*_m_*, ψ*_n_*), is expressed as

2 $$E_{S}\left(\phi_{m},\psi_{n}\right)=\sum_{\mu =-k}^{k}\sum_{\nu =-l}^{l}F\left(\mu ,\nu \right)e^{2\pi i\cdot \left(\frac{\mu \cdot m}{M}+\frac{\nu \cdot n}{N}\right)}$$

where *M* and *N* are the total number of grid points along the φ and ψ angles, respectively. The Fourier transform is truncated to 2*k* + 1 terms in the φ direction and 2*l* + 1 terms in the ψ direction. The subscript *S* in *E_S_* (ϕ_*m*_, ψ_*n*_) indicates that the energy correction surface has been smoothed by discarding higher-order terms in the Fourier transform.

In eq 2, *F*(μ, ν) represents the Fourier transform of the energy correction used in traditional grid-based CMAP, *E*(ϕ_*m*_, ψ_*n*_).

3 $$F\left(\mu ,\nu \right)=\frac{1}{M\cdot N}\sum_{m=0}^{M-1}\sum_{n=0}^{N-1}E\left(\phi_{m},\psi_{n}\right)e^{-2\pi i\cdot \left(\frac{\mu \cdot m}{M}+\frac{\nu \cdot n}{N}\right)}$$

Although eqs 3 and 2 may appear to break the symmetry between the Fourier transform and its inverse, this is not the case. The fact that $e^{-2\pi i\left(\mu \cdot \frac{M-1}{M}\right)}$ equals $e^{2\pi i\left(\mu \cdot \frac{1}{M}\right)}$ for any integer value of μ ensures consistency. Specifically, when *k* and *l* are chosen to be *M*/2 and *N*/2, respectively, *E_S_* (ϕ_*m*_, ψ_*n*_) is identical to *E*(ϕ_*m*_, ψ_*n*_).

The primary advantage of SFT-CMAP over traditional grid-based CMAPs is the reduced overfitting achieved by eliminating higher-order terms in the Fourier transform. These higher-order terms reflect fast spatial variations, which are often caused by deficiencies in the force field modeling of short-range repulsion. In contrast, torsional forces are generally believed to originate from bond dipoles or orbital interactions across the central bond,^60,61^ suggesting they should be slow-varying. Consequently, SFT-CMAP provides a clearer separation between torsional interactions and steric hindrance.

The SFT-CMAP approach also leads to a significant reduction in the number of parameters required. For example, a typical 24 × 24 CMAP has nearly four times as many parameters as a 6 × 6 SFT-CMAP, which is used in this work. Furthermore, traditional grid-based CMAPs do not strictly enforce smoothness of the PES across boundaries. Specifically, conformations with torsional angles of 180° are equivalent to those with angles of −180°. Although traditional CMAPs may not exhibit discontinuity issues at boundaries in practice, SFT-CMAP mathematically eliminates any possibility of jumps across the boundary, ensuring a smooth and continuous PES.

We note that the *E*(ϕ_*m*_, ψ_*n*_) being fit cannot be the difference between the gas-phase MP2 surface and the model surface,^8^ as the model was fitted against a QM/MM reference with the peptide fully surrounded by water molecules. In fact, the gas phase peptide MP2 dipole moments are only approximately 60% the dipole moments of the AFM model.

To address this issue, we fit *E*_pep_, which is the peptide-only energy obtained from CPCM calculations according to eq 1. However, the CPCM dipole moment is still roughly 20% smaller than the model dipole moment in the most thermodynamically important regions of the conformational space. Therefore, we scaled the atomic charges of the amide by a factor of 1/1.2 when evaluating the PES of the model to ensure a compatible charge model. We note that this scaling was only applied to regions outside of φ of −15° to 15°, as scaling within this range leads to unbalanced short-range repulsion and Coulombic interactions between CO and NH groups in close contact.

The SFT-CMAP was fitted against the difference between *E*_pep_ and the model energy with scaled charges. We truncated the SFT-CMAP to the sixth order, resulting in an SFT-CMAP with 169 real parameters. This is significantly fewer than the 576 parameters in a traditional 24 × 24 CMAP. Additional details of the SFT-CMAP fitting procedure can be found in the Supporting Information.

The final alanine model, which incorporates the 6 × 6 SFT-CMAP correction, will be referred to as the ALAMP2_25 model. The force field parameters of the model are provided in the Supporting Information. The GROMACS input files for various peptides simulated are also provided.

## 3 Results and Discussion

### 3.1 Performance of the Model

To validate the performance of the ALAMP2_25 model, MD and Replica-Exchange-Molecular Dynamics (REMD)^62–65^ simulations were performed for the blocked dipeptide, cationic Ala_3_^+^, Ala_5_^+^, Ala_7_^+^ and cyclic NMe­(1,5) and NMe­(1,6). The water was modeled with the BLYPSP-4F potential, which was an AFM model developed by fitting to post-Hartree–Fock quality reference forces computed using the DFT-Supplemental Potential (SP) approach.^41^ Details of the simulations are provided in the Supporting Information.

The PES of ALAMP2_25 is shown in Figure 4a, and the free energy surface computed by simulating the block-dipeptide in a box of 260 water molecules is shown in Figure 4b. The PES of the ALAMP2_25 model is in good agreement with the peptide-only MP2 surface shown in Figure 2b, indicating the SFT-CMAP correction improves the PES as expected. While the PES shows the C7 region (−75, 90) to be most stable, followed by the β strand (−165, 165), the global minimum in the free energy surface is PP-II (−60, 150). This difference has been attributed to variations in the peptide-water energy in our previous work.^31^

**Figure 4:**
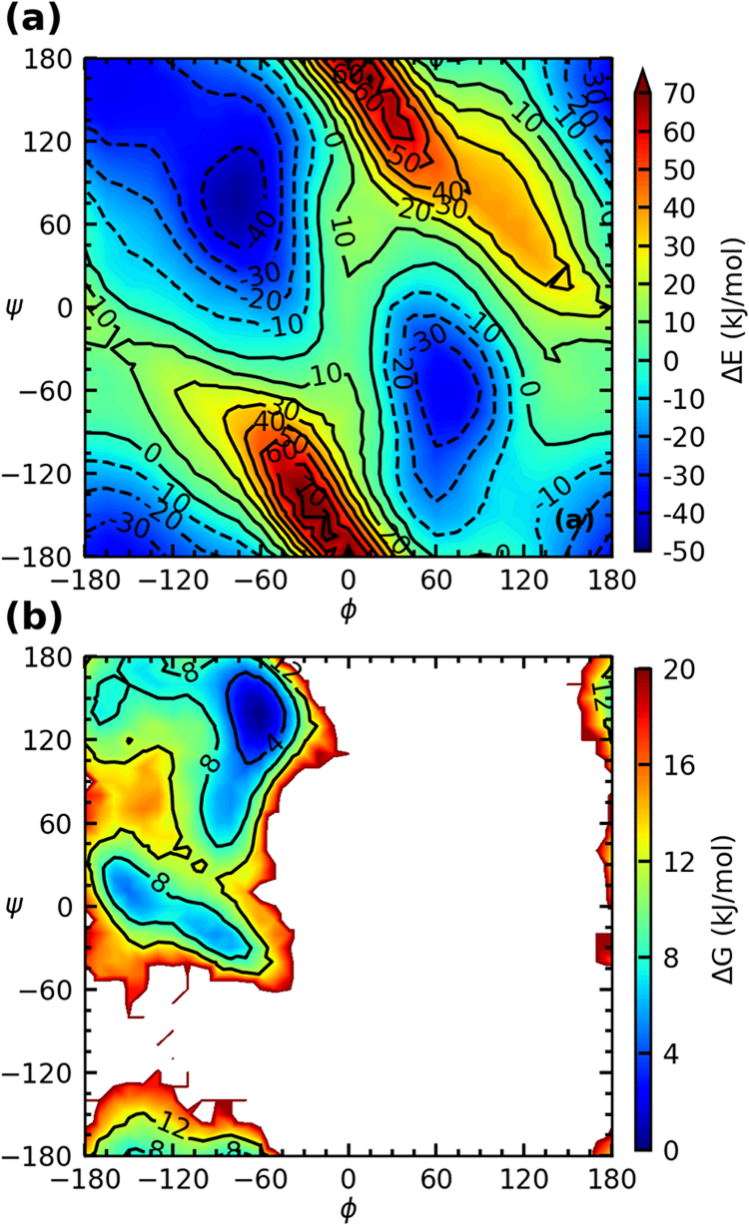
(a) PES of the dipeptide calculated using the ALAMP2_25 model; (b) free energy surface of the blocked dipeptide in aqueous solution.

Although the BLYPSP-4F water model^41^ is nonpolarizable, water still functions as a dielectric medium that can be polarized rotationally by aligning its dipole with an external field, thereby stabilizing peptide conformations with larger dipole moments. Table 1 presents the peptide-water energies and relative energies of various secondary structures for the blocked alanine dipeptide. These energies were computed using restrained MD simulations at each conformation of interest. The peptide-water energies were obtained using the procedure developed by Yuan and Wang,^31^ and details of this procedure are summarized in the Supporting Information. It is worth noting that the water–water energy is expected to increase as water molecules align to stabilize an external dipole. This water–water energy represents the polarization self-energy. When this contribution is considered, the actual stabilization of the peptide by water is approximately half of the raw peptide-water energy.^55^

**Table 1:** Peptide-Water Energies for Blocked Dipeptide before (*E*_p‑w_) and after (*E*_p‑ww_) the Water–Water Polarization Self-Energy Is Considered^a^

		ALAMP2_25		ALA2022	
conformation	φ,ψ (°)	*E*_p‑w_ (kJ/mol)	*E*_p‑ww_ (kJ/mol)	*E*_*p‑w*_ (kJ/mol)	*E*_p‑ww_ (kJ/mol)
α-helix	–60, −45	–238.4(0.0)	–119.2(0.0)	–304.1(0.0)	–152.1(0.0)
β-strand	–135, 135	–171.8(66.6)	–85.9(33.3)	–232.8 (71.3)	–116.4(35.7)
PP-II	–75, 150	–194.9(43.5)	–97.5(21.8)	–269.0 (35.1)	–134.5(17.6)

a The relative energies are shown in parentheses with α helix set to zero.

As shown in Table 1, the peptide-water energy of the MP2-based ALAMP2_25 model stabilizes the α helix conformation over both the β strand and PP-II, similar to what was previously shown with the DFT based ALA2022 model. Furthermore, the β strand is substantially less stable than PP-II due to dielectric frustration,^31^ which arises from the trapezoidal arrangement of the two adjacent dipoles in β strand, making it challenging for water to provide stabilization.

When compared to the ALA2022 model,^31^ the peptide-water energy of the ALAMP2_25 model is 20% weaker, consistent with the expectation of DFT overestimating charge delocalization. Since this reduction in peptide-water energy is not uniform across conformations, the relative energy of different conformations did not decrease uniformly. While both models stabilize the α helix relative to the β strand to approximately the same extent, the relative energy between the β strand and PP-II changed appreciably. The ALA2022 model predicts an 18 kJ/mol penalty for the β conformation relative to PP-II, while this energy difference is reduced to 12 kJ/mol in the ALAMP2_25 model.

Overall, the reduced peptide-water interaction results in the ALAMP2_25 model still showing a predominant PP-II conformation, similar to the ALA2022 model. However, with the peptide-water energy stabilizing the α-helix more and destabilizing the β strand less, the ALAMP2_25 model reduces the penalty for both α helices and β strands relative to PP-II in an aqueous environment.

Graf et al. reported experimental *J*-coupling constants for several cationic polyalanines.^39^ To test the robustness of borrowing parameters for an AFM-based model, we constructed models for cationic Ala_3_^+^, Ala_5_^+^, and Ala_7_^+^ by taking the N-terminus ammonium parameters from the zwitterionic ALA2022 model^31^ and the carboxylic acid parameters from the benzoic acid group of mefenamic acid, which was fitted to B3LYP-D3­(BJ)^66–69^ using the Simple Overlapping Region Method for Force Matching (SORForM).^70^ While borrowing parameters from a carboxylic acid in an aromatic system may involve some approximation, utilizing such parameters only for a terminal group is unlikely to introduce significant errors.

It is worth noting that since AFM models do not use combining rules,^71–74^ borrowing parameters for a certain group involves reusing the partial charges and pair-specific cross-terms between the group and other parts of the molecule being fit. More details of the parameter borrowing procedure are provided in the Supporting Information. The workflow for parameter borrowing has been automated with a series of tools as part of the AFMTools package.

Figure 5 shows the free energy surface of the Ala_3_^+^, Ala_5_^+^, and Ala_7_^+^ with the *J*-coupling constants summarized in Tables 2, 3 and 4. The χ^2^ value, indicating the agreement with experiments,^75^ is shown in Table 5. The definition of χ^2^ and the details for the *J*-coupling constants calculations are included in the Supporting Information.

**Table 2:** *J*-Coupling Constants for Ala_3_^+^

	*J*-coupling constants/Hz		
residue	type	ALAMP2_25	Exp
A2	^3^J(H_N_,H_α_) (ϕ_2_)	5.52	5.68
	^3^J(H_N_,C′) (ϕ_2_)	1.37	1.13
	^3^J(H_α_,C′) (ϕ_2_)	1.55	1.84
	^3^J(HN,C_β_) (ϕ_2_)	1.95	2.39
	^1^J(N,C_α_) (ψ_2_)	10.97	11.34
	^2^J(N,C_α_) (ψ_2_)	8.23	8.45

**Table 3:** *J*-Coupling Constants for Ala_5_^+^

	*J*-coupling constants/Hz		
residue	type	ALAMP2_25	Exp
A2	^3^J(H_N_,H_α_) (ϕ_2_)	5.53	5.59
	^3^J(H_N_,C′) (ϕ_2_)	1.39	1.13
	^3^J(H_α_,C′) (ϕ_2_)	1.53	1.85
	^3^J(H_N_,C_β_) (ϕ_2_)	1.93	2.30
	^1^J(N,C_α_) (ψ_2_)	11.00	11.36
	^2^J(N,C_α_) (ψ_2_)	8.21	8.55
A3	^3^J(H_N_,H_α_) (ϕ_3_)	5.56	5.74
	^3^J(H_α_,C′) (ϕ_3_)	1.59	1.86
	^3^J(H_N_,C_β_) (ϕ_3_)	2.00	2.24
	^1^J(N,C_α_) (ψ_3_)	10.99	11.27
	^2^J(N,C_α_) (ψ_3_)	8.23	8.40
	^3^J(H_N_,C_α_) (ϕ_3_, ψ_2_)	0.61	0.68
A4	^3^J(H_N_,H_α_) (ϕ_4_)	5.59	5.98
	^3^J(H_N_,C′) (ϕ_4_)	1.33	1.15
	^3^J(H_α_,C′) (ϕ_4_)	1.61	1.89
	^3^J(H_N_,C_β_) (ϕ_4_)	1.95	2.14
	^1^J(N,C_α_) (ψ_4_)	10.94	11.25
	^1^J(N,C_α_) (ψ_4_)	8.20	8.27
	^3^J(H_N_,C_α_) (ϕ_4_, ψ_3_)	0.62	0.69

**Figure 5:**
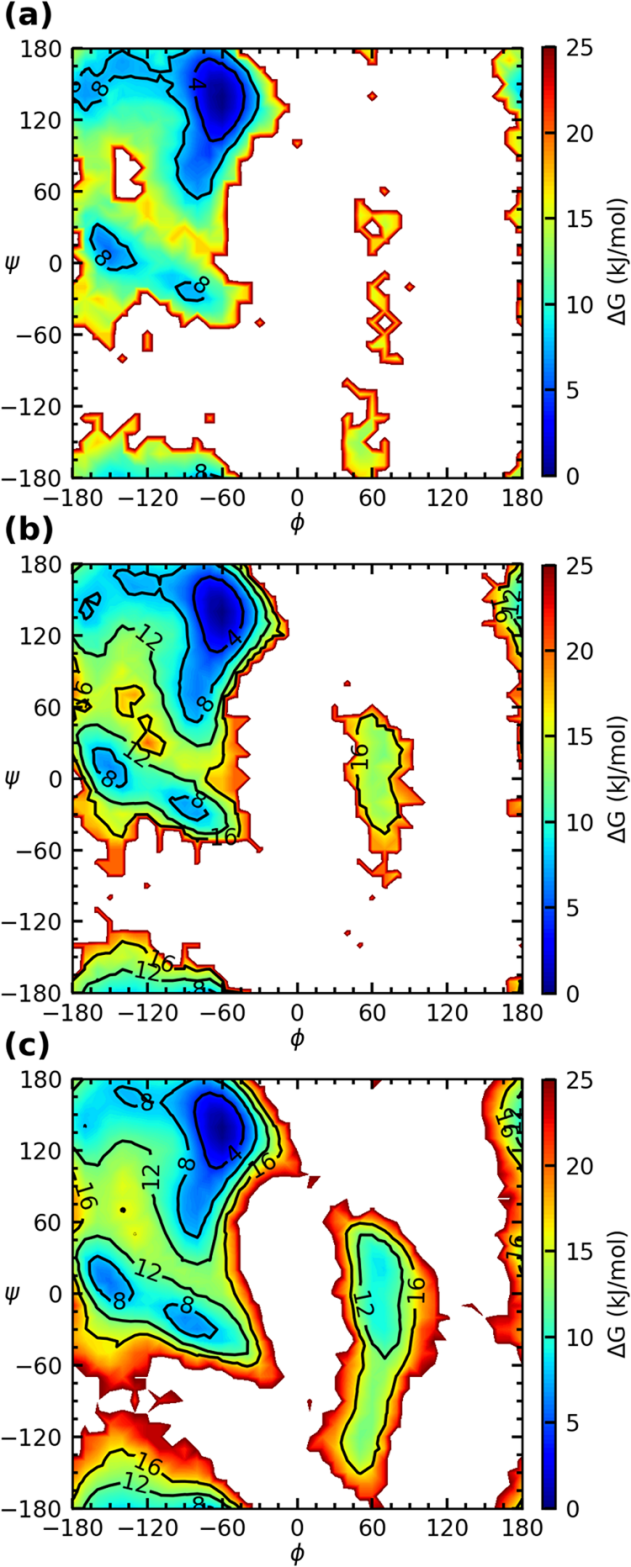
Free energy surface of (a) Ala_3_^+^, (b) Ala_5_^+^ and (c) Ala_7_^+^.

**Table 4:** *J*-Coupling Constants for Ala_7_^+^

	*J*-coupling constants/Hz		
Residue	type	ALAMP2_25	Exp
A2	^3^J(H_N_,H_α_) (ϕ_2_)	5.46	5.61
	^3^J(H_N_,C′) (ϕ_2_)	1.40	1.15
	^3^J(H_α_,C′) (ϕ_2_)	1.53	1.89
	^3^J(HN,C_β_) (ϕ_2_)	1.95	2.31
	^1^J(N,C_α_) (ψ_2_)	10.99	11.37
	^2^J(N,C_α_) (ψ_2_)	8.19	8.52
A3	^3^J(H_N_,H_α_) (ϕ_3_)	5.38	5.66
	^3^J(H_N_,C′) (ϕ_3_)	1.35	1.20
	^3^J(H_α_,C′) (ϕ_3_)	1.59	1.85
	^3^J(HN,C_β_) (ϕ_3_)	2.03	2.20
	^1^J(N,C_α_) (ψ_3_)	10.92	11.27
	^2^J(N,C_α_) (ψ_3_)	8.19	8.29
	^3^J(H_N_,C_α_) (ϕ_3_, ψ_2_)	0.60	0.66
A4	^3^J(H_N_,H_α_) (ϕ_4_)	5.59	5.77
	^3^J(H_N_,C′) (ϕ_4_)	1.36	1.20
	^3^J(H_α_,C′) (ϕ_4_)	2.04	1.80
	^3^J(HN,C_β_) (ϕ_4_)	1.94	2.23
	^1^J(N,C_α_) (ψ_4_)	10.83	11.22
	^2^J(N,C_α_) (ψ_4_)	7.99	8.22
	^3^J(H_N_,C_α_) (ϕ_4_, ψ_3_)	0.60	0.56
A5	^3^J(H_N_,H_α_) (ϕ_5_)	5.87	5.92
	^3^J(H_N_,C′) (ϕ_5_)	1.29	1.19
	^3^J(H_α_,C′) (ϕ_5_)	2.02	1.56
	^3^J(HN,C_β_) (ϕ_5_)	1.87	2.23
	^1^J(N,C_α_) (ψ_5_)	10.82	11.29
	^2^J(N,C_α_) (ψ_5_)	7.86	8.24
A6	^3^J(H_N_,H_α_) (ϕ_6_)	5.81	6.04
	^3^J(H_N_,C′) (ϕ_6_)	1.32	1.10
	^3^J(H_α_,C′) (ϕ_6_)	1.71	1.67
	^3^J(HN,C_β_) (ϕ_6_)	1.86	2.21
	^1^J(N,C_α_) (ψ_6_)	10.82	11.29
	^2^J(N,C_α_) (ψ_6_)	8.00	8.18

**Table 5:** χ^2^ of Hydrated Polyalanine for Different Models^a^ ^31^

	Ala_3_	Ala_5_	Ala_7_
ALAMP2_25	1.18	0.75	0.83
ALA2022	0.62	0.62	0.52
ff19SB	1.87	1.18	1.24
C36m	2.36	1.02	1.10

a The ALAMP2_25 values were calculated with cationic peptides in this work. The ALA2022, ff19SB and C36m were for zwitterionic peptides taken from literature.^31^

The free energy surfaces of the three cationic peptides are rather similar to each other and also similar to that of the blocked dipeptide shown in Figure 4b, indicating that the headgroup only plays a minor role in the conformational distribution of these peptides. It is worth noting that, although distribution was collected for five pairs of φ, ψ angles for Ala_7_^+^, only one pair was used for Ala_3_^+^. Consequently, the increased area in the Ramachandran plot with no data for shorter peptides does not indicate a difference in the free energy surfaces.

The individual *J*-coupling constants in Tables 2–4 show quantitative agreement with experimental values, with a maximum deviation of less than 0.4 Hz. The χ^2^ value obtained with the ALAMP2_25 model is close to that reported previously for ALA2022 zwitterionic peptides, and lower than those of either CHARMM C36m^7^ or AMBER ff19SB.^8^ We note that the computation of *J*-coupling constants requires empirical parameters within the Karplus equations;^76–79^ consequently, the slightly better agreement of ALA2022 could be fortuitous. A χ^2^ value not much larger than one is generally considered indicative of good agreement.^80^

### 3.2 Transferability to *N*-Methylated Cyclic-Peptides

If the models developed are truly devoid of error cancellations, one would expect the parameters fitted for linear peptides to accurately describe cyclic peptides, those fitted for l-amino acids to describe the d-isomers, and the ability to seamlessly transfer functional groups between different models. Taking advantage of the available NMR *J*-coupling constants and distance restraints for *N*-methylated cyclic alanine,^40^ we will construct models for two *N*-methylated cyclic peptides, NMe­(1,6) and NMe­(1,5), using the ALAMP2_25 parameters and assess whether the models provide reasonable agreement with the experimental NMR data. Although it is straightforward to fit these peptides using AFM, we deliberately choose not to do so, as the primary objective of this exercise is to evaluate the transferability of the models.

While the details of constructing the cyclic NMe models are described in the Supporting Information, we highlight here the key considerations in building these models. The methyl group on the nitrogen atom will borrow parameters from the side-chain methyl group. However, whereas the side-chain methyl is neutral, the hydrogen substituted by the methyl group has a positive charge of 0.32e. We decided to assign this 0.32e charge to the carbon atom, as the torsional terms are expected to couple with the 1–4 Coulombic interactions. Although this approach does not preserve the 1–4 Coulombic interactions exactly, due to the difference in N–C and N–H bond lengths, it introduces minimal perturbation to the model. Another important aspect to note is that both NMe­(1,5) and NMe­(1,6) contain a d-alanine residue. To utilize the CMAP potential fitted for l-alanine for the d-alanine residue, an inversion operation must be applied, wherein φ is mapped to −φ and ψ is mapped to –ψ.^81^ For comparison, cyclic NMe models for ALA2022, Amber ff19SB, and CHARMM C36m were constructed following a similar approach with additional details provided in the Supporting Information.

For each peptide, there are 35 to 37 NMR distance restraints, but only four ^3^ *J* coupling constants,^40^ which is fewer than one *J*-coupling constant per residue. It is likely more informative to use the distance restraints to evaluate model performance. We compute the Root Mean Square Distance Violation (RMSD_vio_) according to the formula

4 $$\mathrm{RMS}D_{\mathrm{vio}}=\sqrt{\frac{\sum d_{\mathrm{vio}}^{2}}{N_{\mathrm{cons}}}}$$

where *d*_vio_ is the absolute deviation of the average distance measured in MD from the NMR constraints, and *N*_cons_ is the total number of distance constraints available from NMR. Table 6 reports the performance of each model in reproducing the NMR constraints.

**Table 6:** RMSD_vio_ in Å for Various Models

model	NMe(1,5)	NMe(1,6)	overall
ALAMP2_25	0.20	0.29	0.25
ALA2022	0.89	0.51	0.70
ff19SB	0.40	0.15	0.28
C36m	0.35	0.23	0.29

For the NMe­(1,6) cyclic peptide, Amber ff19SB yields the smallest RMSD_vio_, followed by CHARMM C36m and the ALAMP2_25 model. All three models exhibit an RMSD_vio_ less than 0.3 Å, which indicates good agreement. ALA2022 performed the worst, showing a larger error of 0.51 Å. The performance of ALAMP2_25 in predicting the structure of NMe­(1,5) is particularly noteworthy. The model yields an RMSD_vio_ of 0.2 Å. The NMR reference structure obtained with Distance-bound Driven Dynamics (DDD)^40,82,83^ gave a RMSD_vio_ of 0.23 Å, which is not as good as prediction from the ALAMP2_25 model without using DDD. Furthermore, the ALAMP2_25 model gives lower RMSD_vio_ than both CHARMM C36m and AMBER ff99sb for this peptide. The ALA2022 model produced the worst performance. NMe­(1,5) contains a cis-peptide bond, which is preserved in simulations using the ALAMP2_25, Amber ff19SB and CHARMM C36m models. The ALA2022 model equilibrated it to a trans-peptide bond.

When considering both cyclic peptides, the MP2-based ALAMP2_25 model produced good agreement with experimental data, yielding the lowest average RMSD_vio_ of all the models. Both the recent CHARMM and AMBER models performed very well, with ALA2022 exhibited some deficiencies. Recent structural analysis^84^ shows that the cyclic peptides have type I β-turns (φ_i_ = −60, ψ_i_ = −30, φ_i+1_ = −90, ψ_i+1_ = −0), which may be too high in energy with the ALA2022 model. The most likely cause for this deficiency is that the ALA2022 model lacks explicit modeling of φ and ψ coupling and the model overstabilizes the PP-II conformation due to overestimation of peptide-water interactions.

## 4 Summary and Conclusions

In summary, a novel force field model for polyalanine (ALAMP2_25) was developed with AFM through fitting to model compounds. Compared with traditional AFM models, this approach leverages the transferability of parameters for molecules in similar environments and allowed the construction of force fields using higher quality reference calculations that are more computationally demanding. As the relative stability of peptide conformations depends strongly on the peptide-water energy, the ability to use MP2 rather than DFT, which has known deficiencies in modeling water, is a major step forward.

Furthermore, a new correction scheme, based on a Fourier Transform (SFT-CMAP), was introduced to efficiently describe φ, ψ coupling. SFT-CMAP dramatically reduces the number of parameters while ensuring a smooth PES and decoupling slow-varying torsional interactions from fast-varying steric effects. We posit that SFT-CMAP effectively models inductive effects between adjacent polar groups along the peptide backbone and provide a correction for imbalanced treatment of dipole–dipole interactions involving 1–3 Coulombic interactions.

Although ALAMP2_25 substantially reduces the contributions of peptide-water energies to the total potential energies of the system, its effect on the relative energies is more limited. When compared to the DFT-based ALA2022 model, the relative energy difference between α-helices and β-strands remained roughly the same, whereas the relative stability of the PP-II conformation was reduced. Overall, ALAMP2_25 achieved similar agreement with experimental *J*-coupling data as the ALA2022 model for cationic polypeptides but allowed a higher population for both α-helices and β-strands.

Notably, the ALAMP2_25 model demonstrates good transferability to *N*-methylated cyclic alanine, achieving competitive or superior agreement with NMR data compared to established force fields without any parameter refitting. This improved transferability compared to the ALA2022 model indicates that the new model better captures the underlying physics responsible for the small differences in conformational stability of polypeptides.

The model-compound-based fitting framework developed in this study, along with the demonstrated transferability of pair-specific cross-terms between different functional groups, provides a pathway for creating accurate force fields for a broader range of amino acids and other types of biomolecules. Future work will focus on expanding this methodology to additional protein residues, DNA, lipids, and carbohydrates.

In conclusion, this work paves the way for future research into *ab initio* based model development, enabling first-principles based simulations of complex biological systems, which have broad application in fields, such as protein folding, ligand binding, and drug design.

## Supplementary Material



## Notes

Detailed procedure for the fragment-based fitting of the ALAMP2_25 model, details of the SFT-CMAP approach, parameters of the ALAMP2_25 model, simulation details for property calculations, detailed procedure for the computation of peptide-water energies, detail of the construction of the models for the cationic peptides by borrowing parameters, determination of NMR scalar J coupling constants and χ^2^, and details of the construction of the models for the *N*-methylated cyclic peptides (PDF)
