# Supplementary material for: An Adaptive Force Matching Potential for Alanine Developed with Møller–Plesset Perturbation Theory and Smooth Fourier Transform Correction Map
Source: J Phys Chem B. 2025 Aug 26;129(36):9165–74. doi: 10.1021/acs.jpcb.5c04529 (PMC12434672; doi:10.1021/acs.jpcb.5c04529)
Supplement: Supplementary file 1 [file jp5c04529_si_001.pdf]

Supporting Information

for

An Adaptive Force Matching Potential for Alanine Developed with Møller–Plesset Perturbation Theory and Smooth Fourier Transform Correction Map

Ying Yuan[1] and Feng Wang[1,*]

[1]Department of Chemistry and Biochemistry,

University of Arkansas,

Fayetteville,

Arkansas, 72701, USA

[*] fengwang@uark.edu

S1. Detailed Procedure for the Fragment-Based Fitting of the ALAMP2_25 Model.

For MP2-based fitting of the alanine model, it is necessary to employ a fragment-based procedure to minimize the size of the Quantum Mechanics (QM) region and assume that parameters fitted with smaller molecules are transferable to larger polypeptides and proteins. In this work, dispersion parameters are fitted using N-Methylacetamide (NMA)-water dimers. Intermolecular interactions between peptides and water are fitted using hydrated dipeptides, and intramolecular peptide terms are fitted using tripeptides.

a. Determination of dispersion parameters

In Adaptive Force Matching (AFM), the dispersion parameters are typically determined prior to AFM iterations because the power law terms used to model dispersion couple strongly with repulsion, leading to parameter stability issues when fitted together.

The dispersions are pair-specific and modeled using a short-range damped dispersion[1] of the form:

$$U_{disp}(r) = \frac{C_6}{r^6 + r_0^6} \tag{S1}$$

where r_0 is 0.6 times the sum of the van der Waals radii[2] of the atoms in the pair. Dispersion interactions are only included between heavy atoms.

For the dispersion between water and the amide group on the backbone, the parameters are fitted against SAPT E2 dispersion[3] computed with the PSI4 program.[4] These calculations were performed using the def2-TZVPD basis set with the dimer-centered basis set (DCBS) approximation. The dispersion is determined by fitting the E2 energies between N-Methylacetamide (NMA) and water. To generate these dimers, 1 ns of MD simulations were

performed in a cubic box containing one NMA molecule and 512 water molecules modeled with the BLYPSP-4F model.

From these simulations, a total number of 2100 dimers were extracted with the nearest-atom distance between NMA and water ranging from 5 to 12 Å with a uniform distribution. This distribution was achieved by dividing the 5 to 12 Å range into 35 windows and ensuring an equal number of conformations in each window.

Since SAPT dispersion is expected to be only weakly dependent on monomer geometry, each dimer was optimized for 5 steps at the Hartree-Fock level using the same basis set as used for SAPT. Selected intermolecular distances were restrained to prevent changes in molecular spacing and orientations as the monomer geometry approached the HF minimum.

The dispersion parameters for interactions between the side-chain methyl groups and between the side-chain methyl group and water were taken from the MP2-based pure ethane and hydrated ethane models.[5] All other dispersion parameters were taken from ALA2022 without refitting.[6]

Following the determination of the dispersion parameters, AFM iterations will be carried out holding these parameters fixed at their predetermined values. The AFM generations will iterate over intermolecular fits and intramolecular fits, with intermolecular fits performed before intramolecular fits.

b. Dipeptide-based fit of intermolecular parameters

The intermolecular peptide-water interactions, including partial charges and short-range non-bonded terms, are fitted using the blocked dipeptide, Ace-Ala-NMe, in water during each generation of AFM. The atom types are shown in Figure 1a of the main text.

In the MD step, all simulations were performed using GROMACS[7] at 310 K and 1 bar with a dipeptide in a cubic box containing 1399 water molecules. Hydrogen mass is replaced by that of the tritium to allow for a 1 fs time step size. The temperature was controlled using a stochastic rescaling thermostat[8] with a 1 fs time constant, and the pressure was maintained using the Berendsen barostat with a 0.5 ps time constant. Each trajectory had a simulation length of 1 ns.

The sampling was performed with the ϕ and ψ torsional angles constrained to one of the four groups, as shown in Table S1. The first three groups represent important regions typically sampled in proteins, while the survey group covers a wide range of less frequently sampled conformations to ensure that no artifacts emerge in the potential energy surface (PES). These constraints were applied using a cosine function with an amplitude of 20 kJ/mol and m=1. The constrained conformations were extracted from the end of each trajectory with a 10 ps spacing between frames.

Table S1. Constrained dihedral angles and number of conformations (*N*) for each conformational group used for sampling.

Group	ϕ ,ψ (°)	*N*
C5/beta	(-150,150)	60
PPII	(-75,150)	60
Alpha helix	(-60,-45)	60
Survey Group	(60,45)	20
	(-80,80)	20
	(80,-80)	20
	(-90,-135)	20
	(-150,-60)	20
	(-150,0)	20
	(-150, 60)	20
	(-49,-36)*	20

*This last constraint is only used when fitting intramolecular terms to improve the sampling of a few intramolecular repulsions. (see discussion in Sec S1.c)

For each conformation in the training set, a QM/MM calculation is performed using the density-fitting MP2 method[9] for the QM region, with the MM region modeled using Coulombic embedding. The QM region is further divided into a fitting zone and a buffer zone, where only forces on atoms in the fitting zone are used for AFM.

The QM and MM regions were identified using the following steps:

(a) The dipeptide was placed in the fitting zone, and any water molecules within 3.2 Å of the peptide were included in the QM region.

(b) Five randomly selected QM water molecules were added to the fitting zone. Any water molecules within 2.6 Å of these five waters will be added to QM region.

(c) Water molecules within 6.0 Å of any QM atom were retained in the MM region, while all other water molecules were discarded.

(d) To maximize the number of waters to be fitted, any water molecule without an MM particle within 2.6 Å was included in the fitting zone. The remaining molecules in the QM region outside of the fitting zone were assigned to the buffer zone.

This procedure results in a QM region containing between 27 and 48 QM water molecules, depending on the conformation. The average number of water molecules in the fitting zone is approximately six.

Reference forces were calculated with QM/MM using density fitting MP2 with the def2-TZVP basis set for fitting zone atoms and the def2-SVP basis set for buffer zone atoms. MM water was modeled using the BLYPSP-4F water model. All QM/MM calculations were performed using ORCA.[10]

In the FM step, partial charges were only assigned to the amide atoms (atom types C2, N1, O1, and H2 in Figure 1a), while none of the other peptide atoms had Coulombic interactions. As AFM fits linear parameters whenever possible, it does not directly fit atomic charges; instead, partial charges were deduced from the product of charges.[11, 12] In this work, the atomic charges for the amide group were obtained from charge products with water molecules in the fitting zone. The amide group was constrained to be neutral with a weight of 1000.

The short-range repulsion terms between atoms of types *i* and *j* were modeled using an exponential function:

$$V_{rep}(r_{ij}) = A_{ij} e^{-\alpha_{ij} r_{ij}} \quad \text{(S2)}$$

where r_{ij} is the distance between the two atoms, and α is fitted with a harmonic constraint of:

$$P(\alpha_{ij}) = w_p (\alpha_{ij} - \alpha_0)^2 \quad \text{(S3)}$$

The restraint α_0 was set to 3.5 $Å^{-1}$, and the weight w_p was 1.0. An exponent of 3.5 $Å^{-1}$ was determined to be optimal by fitting the exchange-repulsion component from SAPT calculations for similar atoms.[1] As the model uses more repulsion terms than necessarily, a Harmonic restraint increases parameter stability.

Repulsion terms were placed between every pair of heavy atoms and every pair of atoms that do not already have Coulombic repulsion. The methyl-water parameters were taken from a previously developed model for hydrated ethane[5] without refitting during AFM.

We note that the N1-HW repulsion was not stable in our fit, possibly because the N1 and HW atoms never approach each other closely enough to sample the steric effect. To avoid unphysical attractions between these oppositely charged atoms in extended simulations, we fixed the N1-HW repulsion to 50% of the Amber ff99SB value.[13]

c. Tripeptide-based fit of intra-molecular terms.

After the intermolecular parameters were obtained, the intramolecular terms were fitted using a blocked tripeptide Ace-Ala_2-NMe, as shown in Figure 1b of the main text.

The MD sampling step was performed with the tripeptide in a cubic box containing 880 water molecules. Restraints on ϕ and ψ angles were applied, as specified in Table S1, with the same restraint parameters as those used for the dipeptide. To improve sampling of intramolecular repulsions, which can be challenging, an additional set of trajectories was run at an elevated temperature of 360 K during the last two generations of AFM and included in the global fit.

In the QM/MM step, only the peptide was included in the fitting zone of the QM region. Water molecules within 3.2 Å of fitting zone atoms were included in the buffer zone, while

water molecules within 6.0 Å of the QM region were treated as MM water. The QM method and basis set used were the same as those employed for the intermolecular fit.

In the FM step, the partial charges and all peptide-water terms were fixed to the values obtained in the intermolecular fit. To enhance the fitting accuracy of the backbone atoms, these atoms (C2, O1, N1, H2, C3, and CA) were assigned a weight of 3, while all other atoms had a weight of 1.

The intramolecular short-range repulsion terms were modeled using Eq. 1 with an exponential function α fixed at 3.5 $Å^{-1}$. If two atoms never came into contact, which was defined as being closer than 3.5 Å between two heavy atoms or closer than 3.0 Å if at least one of the atoms was hydrogen, there was no information in the training data to fit a short-range repulsion. In such cases, Amber ff99SB repulsions[13] were added between heavy atoms.

The bond terms were modeled using a harmonic bond potential, and the angle terms were modeled using harmonic angles. For the angles surrounding the C2 and N1 atoms, the equilibrium angles were fixed to the optimized values using MP2/def2-TZVP for NMA, as summarized in Table S2.

Table S2. NMA Angles Used for Fitting the Peptide Model

angle types in the peptide	angles (°)
C3_C2_O1/ C1_C2_O1	122.32
C3_C2_N1/C1_C2_N1	115.86
N1_C2_O1	121.83
C2_N1_C3/C2_N1_C4	120.50
C2_N1_H2/C4_N1_H2	119.90
C2_N1_H2	119.45

The torsional interactions were described using the cosine dihedral formula:

$$V_{dih}(\theta) = k(1+\cos(m\theta-\delta)) \tag{S4}$$

where θ is the torsional angle. The single-bond potential (SP) was fit with a multiplicity *m* of 3 and *δ* of 0° and the double-bond potential (DP) with a *m* of 2 and *δ* of 180°. As discussed previously, only one term is required for single-bond torsional to avoid overfitting.[1] A summary of all the torsional terms used in the force field can be found in Table S3.

Table S3. Fitted torsional terms from tripeptide.

Torsional terms		Fitting-type
ϕ	C2-N1-C3-C2	SP
ψ	N1-C3-C2-N1	SP
ω	C3(C1)-C2-N1-C3(C4)<br>C3(C1)-C2-N1-H2<br>O1-C2-N1-C3(C4)<br>O1-C2-N1-H2	DP
other	C2-C3-CA-HA<br>H1-C1-C2-N1<br>H1-C4-N1-C2	SP

d. Global Fit

A total of seven generations of sampling were completed, and the last four generations were used for the global fit. The global fit included 1280 dipeptide conformations for the peptide-water intermolecular fit, and 2000 tripeptide conformations for the peptide intramolecular fit.

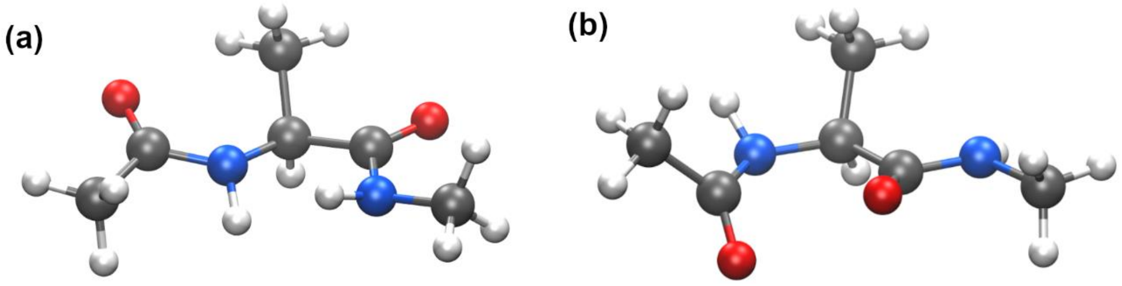

Figure S1. Dipeptide conformations with large energy differences between model and MP2 solute PES. (a) (165,15), (b) (0,-180)

S2. The Smooth-Fourier-Transform (SFT)-CMAP approach

To fit the CMAP using the Smooth Fourier Transform approach, both the MP2 reference surface and the model surface as functions of ϕ and ψ are required. The MP2 energy at each grid point was computed using the conductor-like polarizable continuum model (CPCM) method parameterized for liquid water,[14-18] as implemented in ORCA.

The CPCM energies were computed using gas-phase geometry optimized at each grid point. As discussed in the main text, the peptide energy, E_{pep}, was obtained for each conformation using Eq. 3. The E_{pep} surface is shown as Figure 2(b).

To obtain the energy of the model at each grid point, geometry optimization was performed using GROMACS with conjugate gradient and a force convergence criterion of 0.01 $kJ/mol\cdot nm^{-1}$. The optimization was carried out with harmonic constraints on the ϕ and ψ angles at each grid point, using a force constant of 100,000 $kJ/mol/rad^2$. The resulting model surface is shown as Figure 2(a).

As discussed in the main text, the CPCM dipole moments are smaller than the model dipole moments. To compensate for this, the amide charge was scaled by a factor of 1/1.2.

However, when this surface was used to construct the SFT-CMAP, the resulting model exhibited relatively low energy in the center region, where ϕ is close to zero (Figure S2). This is because the CO-NH groups come into contact and scaling the Coulombic interactions at these conformations lead to an exaggerated repulsion, causing numerical problems with the CMAP. To address this issue, we decided not to scale the charges in the region from ϕ = -15° to ϕ = 15°.

To ensure a smooth model surface with different charge scaling, the region where the partial charge is scaled and the region where it is not scaled are made to match by shifting the unscaled surface by 38 kJ/mol, which is the average energy difference at the boundary of scaled and unscaled regions. The final model surface used to fit the CMAP correction is shown in Figure S3.

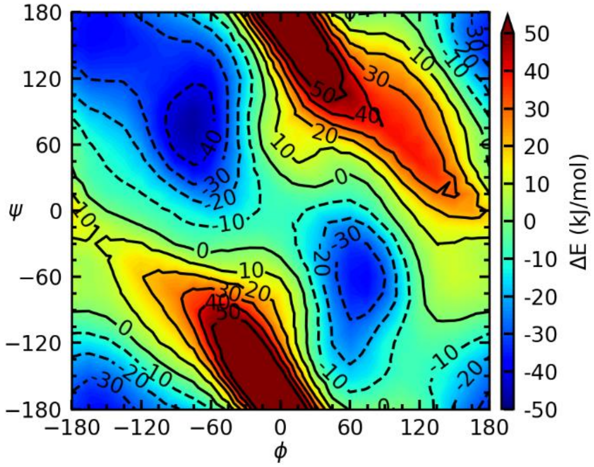

Figure S2. PES with CMAP fitted to the difference between E_{pep} and fully scaled model surface shows an unphysical minimum in the center.

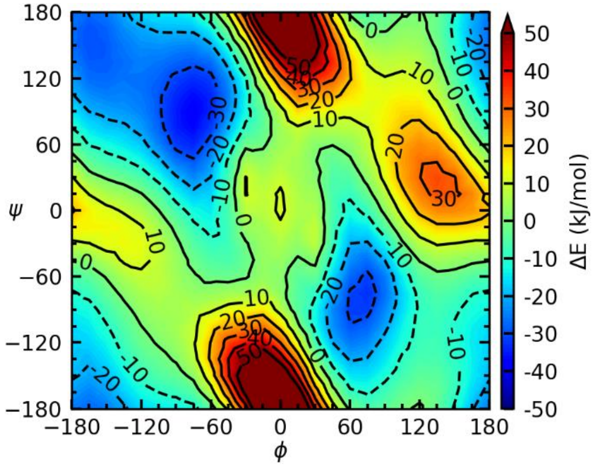

Figure S3. The PES used to fit CMAP, the region from ϕ = -15° to ϕ = 15° is not scaled in this surface.

S3. Parameters of the ALAMP2_25 Model

Parameters for the ALAMP2_25 model are provided in Tables S4 ,S5 and S6, with the CMAP parameters listed in the cmap66.itp file and discussed below.

The GROMACS input files for the blocked dipeptide, $(Ala)_7^+$, NMe(1,5) and NMe(1,6) are provided at https://wanglab.uark.edu/Models.html.

Table S4. Partial charges

Atom type	Partial charges (e)
H2	0.31816
C2	0.26466
O1	-0.46676
N1	-0.11606

Tables S5. Bonded parameters

Bonds	$U=k/2*(r-r_0)^2$	
	r_0(nm)	$k(kJ/(mol\cdot nm^2))$
C2_C3	0.15223885	147002.02
C2_N1	0.13457442	373677.85
C2_O1	0.12344883	600101.06
C3_CA	0.15518773	196693.69
CA_HA	0.10967661	304325.74
C3_N1	0.14690280	240471.83
C4_H1	0.10957784	317401.47
C4_N1	0.14802658	268090.11
C1_C2	0.14904418	181141.67
C1_H1	0.10913035	315067.96
C3_H3	0.11108505	289733.48
H2_N1	0.10134601	421477.57
Angles	$U=k/2*(\Theta- \Theta_0)^2$	
	$\Theta_0(^\circ)$	$k(kJ/(mol\ rad^2))$
HA_CA_HA	111.70096	224.44517
H3_C3_N1	110.18363	400.53168
C2_C1_H1	105.09007	526.61974
C2_N1_C4	120.50000	543.78436
CA_C3_H3	111.75095	356.28719
H1_C1_H1	102.57206	286.10267
C1_C2_O1	122.32000	445.13188
C3_C2_N1	115.86000	344.50603
C2_C3_N1	107.41802	525.68541
C3_CA_HA	114.51092	365.55375
C2_C3_CA	109.49734	494.64475
C2_C3_H3	106.59738	330.55754

N1_C2_O1	121.83000	727.24819	
CA_C3_N1	111.06703	633.83478	
C4_N1_H2	119.90000	244.91496	
C3_N1_H2	119.90000	256.40981	
H1_C4_H1	99.003193	318.01076	
H1_C4_N1	105.24592	501.87176	
C3_C2_O1	122.32000	413.46641	
C1_C2_N1	115.86000	383.56395	
C2_N1_C3	120.50000	644.81905	
C2_N1_H2	119.45000	283.89926	
dihedral	U=A*(1+cos(mϕ-δ))		
	δ(°)	A(kJ/mol)	m
C2_C3_N1_C2	0.0000000	2.4300881	3
N1_C2_C3_N1	0.0000000	-1.1385342	3
C1_C2_N1_C3	180.00000	40.040132	2
C3_C2_N1_C4	180.00000	54.157895	2
C3_C2_N1_C3	180.00000	43.023614	2
C3_C2_N1_H2	180.00000	12.628910	2
C1_C2_N1_H2	180.00000	9.9028444	2
O1_C2_N1_C3	180.00000	44.446777	2
O1_C2_N1_C4	180.00000	49.516314	2
O1_C2_N1_H2	180.00000	18.300664	2
C2_C3_CA_HA	0.0000000	0.30926985	3
H1_C1_C2_N1	0.0000000	-0.40458657	3
H1_C4_N1_C2	0.0000000	0.98356831	3

Table S6. Nonbonded parameters

Energy Expression	Listed as
Exponential: A·exp(-α·r)	EXP A(kcal/mol) α(Å^{-1})
Amber Repulsion (Inverse Power Law): A/r^{12}	POW A(kcal mol^{-1} Å^{12})
Short-range Damped Dispersion: C6/(r^6+r_c^6)+ C8/(r^8+r_c^8)	when C8 is zero: SRD C6(kcal mol^{-1} Å^6) r_c(Å) otherwise: SRD C6(kcal mol^{-1} Å^6) r_c (Å) C8 (kcal mol^{-1} Å^8)

atom1	atom2	expression	parameters	
H1	HW	EXP	449.17425	2.9706969
C4	HW	EXP	421.05978	3.4389765
O1	HW	EXP	2398.1217	4.2219586
N1	HW	POW	1063.0000	
H3	HW	EXP	1331.7144	3.6510885
HA	OW	EXP	5972.9378	3.8442641
CA	OW	EXP	1497305.4	4.6011583
CA	OW	SRD	-1051.5300	1.9970000
HA	HW	EXP	3368.9255	4.3657896
H1	OW	EXP	8209.5830	4.2438378
H2	OW	EXP	1178.1387	3.6229163

C1	OW	EXP	67552.494	3.5136247
C1	OW	SRD	-1051.5300	1.9970000
C2	OW	EXP	4189.8204	3.2242261
C4	OW	EXP	571563.02	4.3998291
C4	OW	SRD	-1051.5300	1.9970000
O1	OW	EXP	167029.07	4.0585361
O1	OW	SRD	-601.74800	1.9560000
N1	OW	EXP	63236.668	3.4567885
N1	OW	SRD	-628.14200	1.9840000
C3	OW	EXP	30664.057	3.1363436
C3	OW	SRD	-670.74600	1.9970000
C2	C2	SRD	-48.807000	2.1210000
C2	O1	EXP	20782.160	3.5000000
C2	O1	SRD	-457.37700	2.0420000
C2	N1	EXP	49960.787	3.5000000
C2	N1	SRD	-584.33900	2.0700000
C2	C3	POW	924824.28	
C2	C1	POW	924824.28	
C2	C1	SRD	-1473.7910	2.0830000
C2	C4	POW	924824.28	
C2	C4	SRD	-1473.7910	2.0830000
O1	O1	EXP	38962.041	3.5000000
O1	O1	SRD	-163.04300	1.9620000
O1	N1	EXP	38480.658	3.5000000
O1	N1	SRD	-406.80500	1.9910000
O1	H2	EXP	986.09972	3.5000000
O1	C3	EXP	31593.620	3.5000000
C1	C3	SRD	-452.67500	2.0030000
O1	H3	EXP	5664.5354	3.5000000
O1	H1	EXP	15856.233	3.5000000
O1	C1	POW	647840.29	
O1	C1	SRD	-825.57500	2.0030000
O1	C4	EXP	39557.775	3.5000000
O1	C4	SRD	-825.57500	2.0030000
N1	N1	EXP	38510.820	3.5000000
N1	N1	SRD	-69.141000	2.0190000
N1	C3	POW	995484.44	
N1	C3	SRD	-1812.4780	2.0320000
N1	H3	EXP	14114.031	3.5000000
N1	H1	EXP	27723.437	3.5000000
N1	C1	POW	995484.44	
N1	C1	SRD	-900.60800	2.0320000
N1	C4	POW	995484.44	
N1	C4	SRD	-900.60800	2.0320000
H2	C3	EXP	1639.4255	3.5000000
H2	H3	EXP	1749.9282	3.5000000
H2	H1	EXP	1103.8996	3.5000000
H2	C1	EXP	8886.9007	3.5000000

C3	C3	POW	1043083.0		
C3	C1	POW	1043083.0		
C3	C1	SRD	-1522.5820	2.0450000	
C3	C4	POW	1043083.0		
C3	C4	SRD	-1522.5820	2.0450000	
C1	C4	SRD	-1283.9710	2.0450000	-12669.064
HA	HA	EXP	1550.2790	3.7140000	
CA	CA	EXP	949562.48	4.0450000	
CA	CA	SRD	-1283.9710	2.0450000	-12669.064
CA	HA	EXP	166971.95	4.9430000	
C2	CA	EXP	72855.816	3.5000000	
C2	CA	SRD	-1473.7910	2.0830000	
O1	CA	EXP	23832.547	3.5000000	
O1	CA	SRD	-825.57500	2.0030000	
N1	CA	EXP	57416.344	3.5000000	
N1	CA	SRD	-900.60800	2.0320000	
C2	HA	EXP	9834.4542	3.5000000	
O1	HA	EXP	5199.2884	3.5000000	
N1	HA	EXP	4576.5882	3.5000000	
H2	HA	EXP	887.25665	3.5000000	
CA	C3	POW	1043083.0		
CA	C3	SRD	-1522.5820	2.0450000	
CA	C1	SRD	-1283.9710	2.0450000	-12669.064
CA	C4	SRD	-1283.9710	2.0450000	-12669.064

The CMAP correction is provided in the released GROMACS input files as cmap66.itp. Following the line listing the five atoms associated with the coupled ϕ, ψ torsional, 576 energy values of the CMAP are listed on the discrete ϕ, ψ grid points with a 15-degree spacing. The ψ angle varies first, with both ϕ and ψ ranging from -180 to 165°.

S4. Simulation Details for Property Calculations

a. Blocked Dipeptide

The hydrated blocked dipeptide simulations were performed with a cubic box containing 391 water molecules at 300 K and 1 bar. The Nosè-Hoover thermostat[19, 20] and Parrinello-Rahman barostat[21] were used to control the temperature and pressure. The simulation length was 52 ns with the last 50 ns used to compute the free energy. Conformations for free energy

calculations were saved every 1 ps. The free energy was plotted using 10° grids. White space indicates the absence of a saved conformation with that ϕ, ψ angle.

b. Cationic Peptides

The cationic peptides $(Ala)_3^+$, $(Ala)_5^+$,$(Ala)_7^+$ were solvated in cubic boxes containing 320, 800, and 1400 water molecules, respectively. The number of water molecules was determined to ensure at least 1 nm of space between the two ends of the peptide across the box. The BLYPSP-4F water model was used. One chloride was placed in the boxes.

To ensure proper sampling, replica exchange molecular dynamics (REMD) simulations were performed. Twelve temperatures in the range from 300 to 380 K were used for $(Ala)_3^+$ and $(Ala)_5^+$, while sixteen temperatures were used for $(Ala)_7^+$. The temperatures used are summarized in Table S7. Exchange attempts were made every 500 fs. The acceptance rate ranges from 20% to 30%. The simulation length for the REMD simulation was 20 ns for $(Ala)_3^+$ and $(Ala)_5^+$, and 100 ns for $(Ala)_7^+$. The first 10% of each REMD trajectory was discarded. Conformations for free energy calculations were saved every 1 ps. The free energy was plotted using 10° grids. White space indicates the absence of a saved conformation with that ϕ, ψ angle.

Table S7. Temperatures used for REMD simulations for the cationic peptides. The unit is *K*.

$(Ala)_3^+$ and $(Ala)_5^+$							
1	300.00	4	318.27	7	338.92	10	362.43
2	305.85	5	324.87	8	346.41	11	371.01
3	311.94	6	331.75	9	354.24	12	380.00
$(Ala)_7^+$							
1	300.00	5	317.84	9	337.94	13	360.76
2	304.27	6	322.64	10	343.37	14	366.95
3	308.66	7	327.59	11	348.98	15	373.36
4	313.19	8	332.68	12	354.77	16	380.00

c. NMe(1,6) and NMe(1,5)

To study the conformation of NMe(1,6) and NMe(1,5), a single peptide was placed in a cubic box containing 343 water molecules. The initial NMe(1,6) and NMe(1,5) structures were constructed by enforcing selected hydrogen bond restraints to make the fold similar to the template structure reported in the study of Beck et al.[22] For NMe(1,5), the peptide bond preceding the 5th residue was set to cis, as discussed by Beck; however, a restraint is not applied to this ω angle. The initial conformation was equilibrated for 1 ns with the hydrogen bond constraints at 300 K and 1 bar. The constraints were then removed for the REMD simulations, which were run with 12 replicas in the temperature range from 300 K to 400 K. The temperatures used for the REMD are summarized in Table S8. The acceptance rates of the exchange between all replicas were approximately 30%.

Table S8. Temperatures used for REMD simulations in Kelvin.

NMe(1,5)/NMe(1,6)							
1	300.00	4	324.49	7	350.97	10	379.62
2	307.95	5	333.08	8	360.27	11	389.67
3	316.11	6	341.91	9	369.82	12	400.00

An additional 100 ps equilibration is done at each temperature without any constraint before the REMD is performed. The mass of tritium is used for hydrogen atoms during the REMD simulations to allow for a 1 fs time step. The temperature in each replica was controlled with a Nosè-Hoover thermostat with a 2 ps relaxation time, and the pressure was controlled with a Parrinello-Rahman barostat with a 5 ps relaxation time. Exchange attempts were made

every 500 fs. A total of 50 ns of REMD was performed, with the final 40 ns of trajectory at the lowest temperature used to compute the $RMSD_{vio}$.[22]

The $RMSD_{vio}$ as described in the text were computed as violation of the average distances to the maximum and minimum values reported by Beck.[22] For methyl hydrogens, one distance was computed for each of the three hydrogens. The distances with regard to the three hydrogens are averaged together. We note this is different from the approach by Beck et al, where a pseudo-atom at the centroid of the three hydrogens are used.

S5. Detailed Procedure for the Computation of Peptide-Water Energies

The ALAMP2_25 peptide-water energies at various secondary structure basins were computed using a blocked alanine dipeptide in a box of 391 water molecules. The backbone dihedrals ϕ and ψ were constrained with harmonic constraints using a force constant of 100 kJ/mol/rad^2. The temperature was controlled at 300 K with the Nosè-Hoover thermostat with a 2 ps relaxation time, and pressure was controlled at 1 bar with a Parrinello-Rahman barostat with a 5 ps relaxation time. A total of 10 ns of restrained MD were performed with the configurations saved every 0.1 ps.

For each saved conformation, the potential energies of the peptide with (E_{sys}) and without water (E_{pep}), and that of the water only (E_{wat}) were recomputed with `mdrun -rerun` in GROMACS. The peptide-water energy was calculated as[6]

$$E_{pep-wat} = E_{sys} - E_{pep} - E_{wat} \quad \text{(S5).}$$

S6. Construction of the Models for the Cationic Peptides by Borrowing Parameters

Experimental J-coupling constants are available for cationic alanine peptides, $(Ala)_3^+$, $(Ala)_5^+$ and $(Ala)_7^+$.[23] However, the model being fit is for blocked peptides, Ace-$(Ala)_n$-NMe. To construct models for cationic peptides, we will borrow specific pair interactions and bonded terms from chemically similar fragments in other AFM models. We use a set of tools (bp tools) developed and released as part of the AFM tools package to accomplish this.

Figure S4 shows the atom types of NH_3^+ and COOH in a cationic $(Ala)_3^+$. When borrowing parameters, three issues must be addressed: partial charges, non-bonded and bonded parameters. Directly using partial charges from donor models can lead to issues with the sum of charges no longer being appropriate.

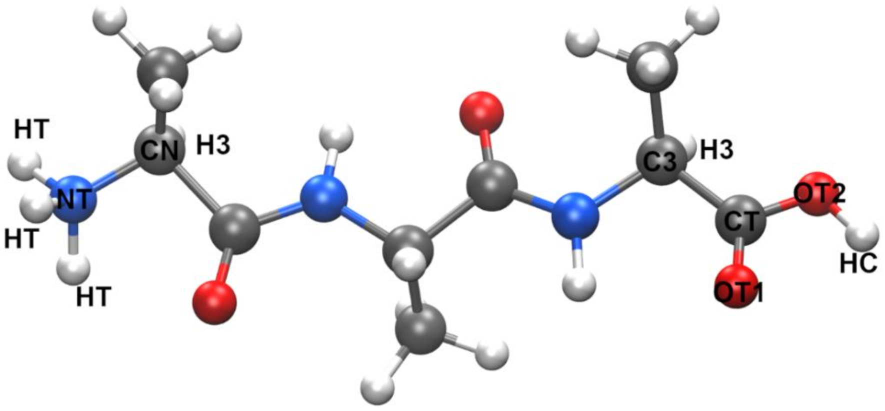

Figure S4. atom types of NH_3^+ and COOH in a cationic $(Ala)_3^+$.

For the NH_3^+ at the N terminus, the partial charges and NH_3^+-water non-bonded interactions are borrowed from the ALA2022 model,[6] which was fit to have a zwitterionic head groups. Since the terminal charge is partially delocalized onto the Cα and Hα in the ALA2022

model, this led to an inappropriate sum of charges. We adjusted the partial charge of the ALA2022 ammonium by adding 0.01009 e to each atom of the group to address this issue.

Similarly, we borrowed the carboxylic acid group at the C-terminus from the aromatic carboxylic group of mefenamic acid.[24] This also led to a charge neutrality problem, which was addressed by adding 0.03073 e to the charge of each atom of the carboxylic acid group. The final charges are summarized in Table S9.

Table S9. Partial charges for NH_3^+ and COOH

Atom type	Charge (e)
NT	-1.11296
HT	0.54965
CN	0.23001
CT	0.63181
OT1	-0.51932
OT2	-0.71240
HC	0.59991

It is worth noting that short-range repulsion terms are expected to be coupled to the partial charges. While small adjustments like those made in this work are not expected to cause problems, any adjustment larger than 0.1 e can be problematic. In other words, we probably should not borrow parameters from a molecular fragment if a substantial adjustment to charges is needed.

For the non-bonded repulsion and dispersion terms between NH_3^+, COOH, with other parts of the peptide, we used the bp (borrow parameter) scripts of AFM tools to borrow from the most similar pairs. The bp tools look for the availability of pair-specific parameters using the

replacement atoms. If the pair-specific term is not available, the corresponding pair-specific parameter between replacement atoms will be used. (See Table S10).

Table S10: Replacement atom types for obtaining the required pair-specific parameters. The model that provides the replacement atom type is in parenthesis.

Atom Type	Replacement Atom Type
NT	N1 (ALAMP2_25)
HT	H2 (ALAMP2_25)
CN	C3 (ALAMP2_25)
CT	C2 (ALAMP2_25)
OT1	O1 (ALAMP2_25)
OT2	OW (ALAMP2_25)
HC	HW (ALAMP2_25)

For example, Figure S4 shows the atom types for a cationic $(Ala)_3^+$. If the CT-C3 pair-specific parameter is not available, the bp scripts will look for the C2-C3 parameters from the ALAMP2_25 model as C2 is the replacement atom for CT.

In general, the bp scripts allow the specification of multiple replacement atom types, each with an associated priority number (PN). The replacement atom type that is a better fit is assigned a lower PN. The script then iterates through a priority list based on the sum of the PNs of each atom in the pair. The first available parameter with the lowest sum of PNs will be used.

The cationic simulation box also contains a counter ion, which is chosen as Cl^-. The Cl^- water non-bonded interactions were taken from our previous work[25] and the Cl^- peptide terms were taken from Amber ff99SB.[13]

A similar procedure was also implemented for borrowing bonded terms. The bonded terms for the termini are shown in table S11.

Table S11. Bonded parameters for cationic terminus.

	Parameters		
bonds	r_0 (nm)	K (kJ/mol·nm^{-2})	
HC_OT2	0.096700955	346736.62	
HT_NT	0.10241055	298093.67	
CN_H3	0.11108505	289733.48	
CT_OT1	0.12211618	638402.59	
CT_OT2	0.13428458	313398.25	
CN_NT	0.14744176	181450.75	
C2_CN	0.15223885	147002.02	
C3_CT	0.14630905	143041.24	
CA_CN	0.15518773	196693.69	
angles	Θ (°)	K (kJ/mol·rad^{-2})	
HT_NT_HT	110.24968	316.12335	
CN_NT_HT	108.42743	364.77272	
H3_CN_NT	103.61069	426.75540	
CA_CN_NT	115.61957	737.17672	
C2_CN_NT	97.997164	513.06648	
CA_CN_H3	111.75095	356.28719	
C2_CN_H3	106.59738	330.55754	
C2_CN_CA	109.49734	494.64475	
CN_CA_HA	114.51092	365.55375	
CN_C2_O1	122.32000	413.46641	
CN_C2_N1	115.86000	344.50603	
CT_C3_N1	107.41802	525.68541	
CT_C3_H3	106.59738	330.55754	
CA_C3_CT	109.49734	494.64475	
C3_CT_OT1	164.95438	417.53453	
C3_CT_OT2	157.44826	340.35589	
OT1_CT_OT2	150.82964	741.55600	
Dihedrals	Θ (°)	K (kJ/mol)	multiplicity
CT_C3_N1_C2	0	2.4300881	3
N1_C2_CN_NT	0	-1.1385342	3
CN_C2_N1_H2	180	12.628910	2
C2_CN_NT_HT	0	0.77379728	3
HC_OT2_CT_C3	0	6.5602623	3

S7. Determination of NMR scalar J coupling constants and χ^2.

With the exception of $^3J(H_N,C_\alpha)$,the J-coupling constants were calculated with the Karplus equation[26] in the form of Eq S6,

$$J(\tau) = A\cos^2(\tau+\theta) + B\cos(\tau+\theta) + C \ , \tag{S6}$$

where τ is the backbone dihedral angle ϕ or ψ. The coefficients *A*, *B*, *C* and offset angle θ were taken from prior experimental fits[27-29] and summarized in Table S12 along with the estimated uncertainty σ_i used to calculate χ^2.

The following equation was used to calculate $^3J(H_N,C_\alpha)$,[30]

$$\begin{aligned} {}^3J_{H_N C_\alpha}(\phi_i,\psi_{i-1}) = & -0.23\cos\phi_i - 0.20\cos\psi_{i-1} + 0.07\sin\phi + 0.08\sin\psi_{i-1} \\ & + 0.07\cos\phi\cos\psi_{i-1} + 0.12\cos\phi\sin\psi_{i-1} \\ & - 0.08\sin\phi\cos\psi_{i-1} - 0.14\sin\phi\sin\psi_{i-1} + 0.54 \end{aligned} \ . \tag{S7}$$

The quality of the agreement with experimental J-coupling data was quantified using χ^2 defined as[31]

$$\chi^2 = \frac{1}{N}\sum_i^N \frac{(\langle J_i \rangle_{sim} - J_{i,\mathrm{expt}})^2}{\sigma_i^2} , \tag{S8}$$

Table S12. Parameters for Karplus equations used in this work.[27-30]

J-coupling	τ	A (Hz)	B (Hz)	C (Hz)	θ (°)	σ (Hz)
$^3J(H_N,H_\alpha)$	ϕ_i	7.09	-1.42	1.55	-60	0.39
$^3J(H_N,C')$	ϕ_i	4.29	-1.01	0.00	180	0.32
$^3J(H_\alpha,C')$	ϕ_i	3.72	-2.18	1.28	120	0.24
$^3J(H_N,C_\beta)$	ϕ_i	3.06	-0.74	0.13	60	0.21
$^1J(N,C_\alpha)$	ψ_i	1.70	-0.98	9.51	0	0.59
$^2J(N,C_\alpha)$	ψ_i	-0.66	-1.52	7.85	0	0.50
$^3J(H_N,C_\alpha)$	ϕ_i, ψ_{i-1}		Eq. S8			0.10

S8. Construction of the Models for the N-methylated Cyclic Peptides

a. ALAMP2_25

The ALAMP2_25 model does not have parameters for the methylated cyclic peptide. To model the N-methylated peptide, we used the side-chain methyl parameters to model the methyl group on the N, which provides all the required cross terms. The atom types are shown in Figure S5. The type H2 hydrogen (Figure 1a) charge was placed on the methyl C (Type CM) bonded to the N1 atom to best preserve the 1-4 Coulombic interactions.

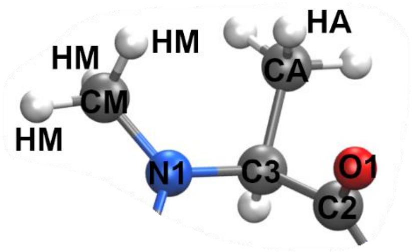

Figure S5. Atom types for N-methylated alanine showing one residue.

The bonded terms related to this methyl group were borrowed from the sidechain methyl group and C terminus of the blocked-trialanine model. For construction of the bonded terms around the N1, H2 is considered the primary substitute atom for CM. The bonded parameters are listed in Table S13.

Table S13. ALAMP2_25 bonded parameters for methyl group bonded to N.

	Parameters in GROMACS		
Bonds	r_0 (nm)	K (kJ/mol·nm^{-2})	
CM_HM	0.10967661	304325.74	
CM_N1	0.14802658	268090.11	
Angles	Θ (°)	K (kJ/mol·rad^{-2})	
HM_CM_HM	111.70096	224.44517	
HM_CM_N1	105.24592	501.87176	
CM_N1_C3	119.90000	256.40981	
CM_N1_C2	119.45000	283.89926	
Dihedrals	Θ (°)	K (kJ/mol)	multiplicity
HM_CM_N1_C2	0	0.98356831	3
CM_N1_C2_O1	180	18.300664	2
CM_N1_C2_C3	180	12.628910	2

As discussed in the main text, for D-alanine, an inversion must be applied to the CMAP by mapping ϕ to -ϕ and ψ to -ψ.

b. ALA2022

The N-methylated peptide model constructed using ALA2022 followed a similar protocol as that of ALAMP2_25 by borrowing the side-chain methyl for the N-methyl. The H2 partial charge was added to the charge on the N-methyl carbon.

The CM_N1 bond, HM_CM_N1 angles and HM_CM_N1_C2 torsional parameters were borrowed from the AFM2021 glycine model,[32] which take NMe as a terminal group. This glycine model was also fitted against B3LYP-D3(BJ) similar to ALA2022.

The final bonded parameters are summarized in table S14.

Table S14. ALA2022 bonded parameters for methyl group bonded to N.

	Parameters in GROMACS		
Bonds	r_0 (nm)	K (kJ/mol·nm^{-2})	
CM_HM	0.10892159	278592.54	
CM_N1	0.14173189	237480.02	
Angles	Θ (°)	K (kJ/mol·rad^{-2})	
HM_CM_HM	104.29271	300.36588	
HM_CM_N1	105.06873	441.98994	
CM_N1_C3	109.88480	265.91800	
CM_N1_C2	113.19431	317.14862	
Dihedrals	Θ (°)	K (kJ/mol)	multiplicity
HM_CM_N1_C2	0	0.96347057	3
CM_N1_C2_O1	180	19.029186	2
CM_N1_C2_C3	180	8.3567150	2

c. Amber ff19SB and CHARMM C36m

For Amber ff19SB and CHARMM C36m, the atom types for the methyl group on the N is the corresponding alkane carbon and hydrogen. For the N-methyl carbon, the charge was similarly adjusted by adding the corresponding hydrogen charge to the carbon. An inversion was applied to the CMAP by mapping ϕ to -ϕ and ψ to -ψ for the D-alanine.

Reference

(1) Yuan, Y.; Ma, Z.; Wang, F. Development and Validation of a DFT-Based Force Field for a Hydrated Homoalanine Polypeptide. *J. Phys. Chem. B* **2021**, *125* (6), 1568-1581.

(2) Anatole von Lilienfeld, O.; Tkatchenko, A. Two- and Three-Body Interatomic Dispersion Energy Contributions to Binding in Molecules and Solids. *J. Chem. Phys.* **2010**, *132* (23), 234109.

(3) Jeziorski, B.; Moszynski, R.; Szalewicz, K. Perturbation Theory Approach to Intermolecular Potential Energy Surfaces of van der Waals Complexes. *Chem. Rev.* **1994**, *94* (7), 1887-1930.

(4) Smith, D. G. A.; Burns, L. A.; Simmonett, A. C.; Parrish, R. M.; Schieber, M. C.; Galvelis, R.; Kraus, P.; Kruse, H.; Di Remigio, R.; Alenaizan, A.; et al. PSI4 1.4: Open-source software for high-throughput quantum chemistry. *J. Chem. Phys.* **2020**, *152* (18), 184108.

(5) Weldon, R.; Wang, F. Exploring the Promise and Limitations of Point-Charge-Free Potentials for Hydrocarbon Modeling. *Scientific Reports* **2025**, *15* (1), 23055

(6) Yuan, Y.; Wang, F. Dipole Cooperativity and Polarization Frustration Determine the Secondary Structure Distribution of Short Alanine Peptides in Water. *J. Phys. Chem. B* **2023**, *127* (14), 3126-3138.

(7) Abraham, M. J.; Murtola, T.; Schulz, R.; Páll, S.; Smith, J. C.; Hess, B.; Lindahl, E. GROMACS: High performance molecular simulations through multi-level parallelism from laptops to supercomputers. *SoftwareX* **2015**, *1-2*, 19-25.

(8) Bussi, G.; Donadio, D.; Parrinello, M. Canonical sampling through velocity rescaling. *J. Chem. Phys.* **2007**, *126* (1), 014101.

(9) Ren, X.; Rinke, P.; Blum, V.; Wieferink, J.; Tkatchenko, A.; Sanfilippo, A.; Reuter, K.; Scheffler, M. Resolution-of-identity approach to Hartree–Fock, hybrid density functionals, RPA, MP2 and GW with numeric atom-centered orbital basis functions. *New J. Phys.* **2012**, *14* (5), 053020.

(10) Neese, F.; Wennmohs, F.; Becker, U.; Riplinger, C. The ORCA quantum chemistry program package. *J. Chem. Phys.* **2020**, *152* (22), 224108.

(11) Akin-Ojo, O.; Song, Y.; Wang, F. Developing ab initio quality force fields from condensed phase quantum-mechanics/molecular-mechanics calculations through the adaptive force matching method. *J. Chem. Phys.* **2008**, *129* (6), 064108.

(12) Nikitin, A.; Wang, F. Simulation of Linear and Cyclic Alkanes with Second-Order Møller–Plesset Perturbation Theory through Adaptive Force Matching. *J. Chem. Theory Comput.* **2024**, *20* (12), 5241-5249.

(13) Hornak, V.; Abel, R.; Okur, A.; Strockbine, B.; Roitberg, A.; Simmerling, C. Comparison of multiple Amber force fields and development of improved protein backbone parameters. *Proteins: Struct., Funct., Bioinf.* **2006**, *65* (3), 712-725.

(14) Klamt, A.; Schüürmann, G. COSMO: a new approach to dielectric screening in solvents with explicit expressions for the screening energy and its gradient. *J. CHEM. SOC. PERKlN TRANS. 2* **1993**, (5), 799-805.

(15) Andzelm, J.; Kölmel, C.; Klamt, A. Incorporation of solvent effects into density functional calculations of molecular energies and geometries. *J. Chem. Phys.* **1995**, *103* (21), 9312-9320.

(16) Barone, V.; Cossi, M. Quantum Calculation of Molecular Energies and Energy Gradients in Solution by a Conductor Solvent Model. *J. Phys. Chem. B* **1998**, *102* (11), 1995-2001.

(17) Cossi, M.; Rega, N.; Scalmani, G.; Barone, V. Energies, structures, and electronic properties of molecules in solution with the C-PCM solvation model. *J. Comput. Chem.* **2003**, *24* (6), 669-681.

(18) Takano, Y.; Houk, K. N. Benchmarking the Conductor-like Polarizable Continuum Model (CPCM) for Aqueous Solvation Free Energies of Neutral and Ionic Organic Molecules. *J. Chem. Theory Comput.* **2005**, *1* (1), 70-77.

(19) Hoover, W. G. Canonical Dynamics: Equilibrium Phase-Space Distributions. *Phys. Rev. A* **1985**, *31* (3), 1695-1697.

(20) Nosé, S. A Molecular Dynamics Method for Simulations in the Canonical Ensemble. *Mol. Phys.* **1984**, *52* (2), 255-268.

(21) Parrinello, M.; Rahman, A. Polymorphic Transitions in Single Crystals: A New Molecular Dynamics Method. *J. Appl. Phys.* **1981**, *52* (12), 7182-7190.

(22) Beck, J. G.; Chatterjee, J.; Laufer, B.; Kiran, M. U.; Frank, A. O.; Neubauer, S.; Ovadia, O.; Greenberg, S.; Gilon, C.; Hoffman, A.; et al. Intestinal Permeability of Cyclic Peptides: Common Key Backbone Motifs Identified. *J. Am. Chem. Soc.* **2012**, *134* (29), 12125-12133.

(23) Graf, J.; Nguyen, P. H.; Stock, G.; Schwalbe, H. Structure and Dynamics of the Homologous Series of Alanine Peptides: A Joint Molecular Dynamics/NMR Study. *J. Am. Chem. Soc.* **2007**, *129* (5), 1179-1189.

(24) Zheng, D.; Yuan, Y.; Wang, F. Fragmentation Method for Computing Quantum Mechanics and Molecular Mechanics Gradients for Force Matching: Validation with Hydration Free Energy Predictions Using Adaptive Force Matching. *J. Phys. Chem. B* **2022**, *126* (16), 2609-2617.

(25) Li, J.; Wang, F. Pairwise-additive force fields for selected aqueous monovalent ions from adaptive force matching. *J. Chem. Phys.* **2015**, *143* (19), 194505.

(26) Karplus, M. Vicinal Proton Coupling in Nuclear Magnetic Resonance. *J. Am. Chem. Soc.* **1963**, *85* (18), 2870-2871.

(27) Wirmer, J.; Schwalbe, H. Angular dependence of $^1J(N_i,C_{\alpha i})$ and $^2J(N_i.C_{\alpha(i-1)})$ coupling constants measured in J-modulated HSQCs. *J. Biomol. NMR* **2002**, *23* (1), 47-55.

(28) Hu, J.-S.; Bax, A. Determination of ϕ and χ1 Angles in Proteins from ^{13}C–^{13}C Three-Bond J Couplings Measured by Three-Dimensional Heteronuclear NMR. How Planar Is the Peptide Bond? *J. Am. Chem. Soc.* **1997**, *119* (27), 6360-6368.

(29) Ding, K.; Gronenborn, A. M. Protein Backbone $^1H^N$–$^{13}C^\alpha$ and ^{15}N–$^{13}C^\alpha$ Residual Dipolar and J Couplings: New Constraints for NMR Structure Determination. *J. Am. Chem. Soc.* **2004**, *126* (20), 6232-6233.

(30) Hennig, M.; Bermel, W.; Schwalbe, H.; Griesinger, C. Determination of ψ Torsion Angle Restraints from $^3J(C_\alpha,C_\alpha)$ and $^3J(C_\alpha,H^N)$ Coupling Constants in Proteins. *J. Am. Chem. Soc.* **2000**, *122* (26), 6268-6277.

(31) Best, R. B.; Buchete, N.-V.; Hummer, G. Are Current Molecular Dynamics Force Fields too Helical? *Biophys. J.* **2008**, *95* (1), L07-L09.

(32) Yuan, Y.; Wang, F. A comparison of three DFT exchange–correlation functionals and two basis sets for the prediction of the conformation distribution of hydrated polyglycine. *J. Chem. Phys.* **2021**, *155* (9),
